# GeoWaVe: geometric median clustering with weighted voting for ensemble clustering of cytometry data

**DOI:** 10.1093/bioinformatics/btac751

**Published:** 2022-11-22

**Authors:** Ross J Burton, Simone M Cuff, Matt P Morgan, Andreas Artemiou, Matthias Eberl

**Affiliations:** Division of Infection and Immunity, School of Medicine, Cardiff University, Cardiff CF14 4XN, UK; Adult Critical Care, University Hospital of Wales, Cardiff and Vale University Health Board, Cardiff CF14 4XW, UK; Division of Infection and Immunity, School of Medicine, Cardiff University, Cardiff CF14 4XN, UK; Adult Critical Care, University Hospital of Wales, Cardiff and Vale University Health Board, Cardiff CF14 4XW, UK; School of Mathematics, Cardiff University, Cardiff CF24 4AG, UK; Division of Infection and Immunity, School of Medicine, Cardiff University, Cardiff CF14 4XN, UK; Systems Immunity Research Institute, Cardiff University, Cardiff CF14 4XN, UK

## Abstract

**Motivation:**

Clustering is an unsupervised method for identifying structure in unlabelled data. In the context of cytometry, it is typically used to categorize cells into subpopulations of similar phenotypes. However, clustering is greatly dependent on hyperparameters and the data to which it is applied as each algorithm makes different assumptions and generates a different ‘view’ of the dataset. As such, the choice of clustering algorithm can significantly influence results, and there is often not one preferred method but different insights to be obtained from different methods. To overcome these limitations, consensus approaches are needed that directly address the effect of competing algorithms. To the best of our knowledge, consensus clustering algorithms designed specifically for the analysis of cytometry data are lacking.

**Results:**

We present a novel ensemble clustering methodology based on geometric median clustering with weighted voting (GeoWaVe). Compared to graph ensemble clustering methods that have gained popularity in single-cell RNA sequencing analysis, GeoWaVe performed favourably on different sets of high-dimensional mass and flow cytometry data. Our findings provide proof of concept for the power of consensus methods to make the analysis, visualization and interpretation of cytometry data more robust and reproducible. The wide availability of ensemble clustering methods is likely to have a profound impact on our understanding of cellular responses, clinical conditions and therapeutic and diagnostic options.

**Availability and implementation:**

GeoWaVe is available as part of the CytoCluster package https://github.com/burtonrj/CytoCluster and published on the Python Package Index https://pypi.org/project/cytocluster. Benchmarking data described are available from https://doi.org/10.5281/zenodo.7134723.

**Supplementary information:**

Supplementary data are available at *Bioinformatics* online.

## 1 Introduction

Clustering is an unsupervised method for identifying structure in unlabelled data. In the context of cytometry, the objective is to categorize events into groups of similar phenotypes. This technique is increasingly being adopted in the field and is widely regarded as an acceptable alternative to manual analysis (Aghaeepour *et al.*, 2013; Cheung et al., 2021; Weber and Robinson, 2016). However, the choice of algorithm appears to be often driven either by its availability in commercial software or ease of its use. In many instances, the reason behind the particular choice of algorithm is not discussed. Of note, clustering algorithms differ in the assumptions made of data, their performance tends to be highly data specific and results can vary widely depending on the chosen hyperparameters (Ghosh and Acharya, 2011; Pedersen and Olsen, 2020; Ronan *et al.*, 2016).

Ensemble clustering (also referred to as consensus clustering) offers an opportunity to reduce this frequently encountered bias by combining the partitions of multiple clustering algorithms run on the same data to identify a consensus that is informed by multiple ‘views’, thereby reducing the dependence on any individual algorithm. Unlike ensemble methods in supervised classification, ensemble clustering has many challenges: the number of clusters may differ amongst the base partitions, the optimal number of consensus clusters is often unknown, and it is necessary to solve the correspondence issue of matching clusters between individual partitions (Boongoen and Iam-On, 2018; Ghosh and Acharya, 2011).

Broadly speaking, ensemble clustering methods can be grouped into three categories: co-association methods, feature-based methods and methods using graph representations (Ghosh and Acharya, 2011; Boongoen and Iam-On, 2018; Vega-Pons and Ruiz-Shulcloper, 2011).

Co-association methods act on the pairwise similarity of clusters sourced from different algorithms. Consensus solutions can be derived from simple techniques such as agglomerative clustering of the binary co-association matrix (*N × N* matrix, where *N* is the number of events, for instance, the number of single cells) (Ronan *et al.*, 2016) or the cluster-based similarity partitioning algorithm (CSPA), that forms partitions on the derived similarity graph using the METIS software (Strehl and Ghosh, 2002). Methods that act on co-association are burdened by space complexity and are therefore intractable for large data where such a matrix exceeds the available computer memory (Ghosh and Acharya, 2011).

Feature-based methods offer an alternative by presenting the problem as a label-association matrix (*m × n* matrix, where *m* is the number of unique clusters). Consensus solutions can be formulated with iterative voting, finite mixture models, pairwise agreement between clusters or agglomerative clustering of this label-association matrix (Boongoen and Iam-On, 2018).

Another popular approach for consensus clustering is by using graph-based methods, where a weighted graph of the clusters contributing to an ensemble is generated and then partitioned into *k* parts using a graph partitioning technique (Ghosh and Acharya, 2011; Boongoen and Iam-On, 2018). Strehl and Ghosh (2002) developed the hyper-graph partitioning algorithm (HGPA) and the meta-clustering algorithm (MCLA), both heuristics that represent the clustering ensemble as a hypergraph. Later the hybrid bipartite graph formulation (HBGF) algorithm was introduced as an alternative approach that models clusters and observations in the same graph. In each case, consensus partitions are constructed from a subsequent bipartite graph (Fern and Brodley, 2004). The advantage of the aforementioned graph methods is their heuristic approach that avoids the need for a co-association matrix, making them applicable to large data.

Ensemble clustering methods have successfully been adopted in the field of single-cell RNA sequencing (scRNA-seq) but the methodologies chosen usually reflect the size of data generated by this technique and do not address the space complexity issues that arise from larger datasets. The graph partitioning-based ensemble method for single-cell clustering, Sc-GPE (Zhu *et al.*, 2020), is an example of a solution deploying co-association to the problem of ensemble clustering, where a co-association matrix is weighted by the similarity (adjusted rand index) of contributing clustering methods. However, the dependence on a co-association matrix makes this technique intractable for cytometry data. The same limitation applies to SC3 (Kiselev *et al.*, 2017), another consensus approach for scRNA-seq employing CSPA for ensemble clustering. Single-cell aggregated (from ensemble) clustering (SAFE-clustering) (Yang *et al.*, 2019) avoids the need for generating a co-association matrix by applying graph-based methods instead but the implementation only allows a limited number of contributing algorithms to the consensus and is exclusively designed for scRNA-seq.

In contrast to these advances in scRNA-seq data analysis, ensemble clustering methods have yet to be developed specifically for cytometry data analysis. Generic techniques from the graph-based ensemble clustering family failed to find additional benefits over existing algorithms (Weber and Robinson, 2016). However, an ensemble methodology that utilizes the label-association matrix showed improved performance compared to individual algorithms (Aghaeepour *et al.*, 2013). Despite the reported improvement, that publication did not disclose a readily available implementation of the methodology, thus making it difficult to reproduce their approach.

Of note, methods developed for scRNA-seq data analysis may not scale to the size of data encountered in cytometry data analysis, which can be hundreds of times greater. We here benchmarked a range of graph ensemble clustering methods against popular clustering algorithms for cytometry data analysis and present a novel ensemble clustering methodology based on geometric median clustering with weighted voting (GeoWaVe). Unlike previous ensemble clustering techniques, GeoWaVe is explicitly designed for cytometry data analysis and offers a computationally inexpensive heuristic approach, permitting the analysis of large data. Compared to graph ensemble clustering methods that have gained popularity in scRNA-seq analysis, GeoWaVe performed favourably on different sets of high-dimensional data generated using cytometry by time of flight (CyTOF) or multicolour flow cytometry. Our findings provide proof of concept for the power of consensus methods to make cytometry data analysis more robust and reproducible.

## 2 Materials and methods

### 2.1 Benchmarking datasets

Six cytometry datasets were chosen for benchmarking ensemble clustering methods (Supplementary Table S1). The public CyTOF datasets ‘*Levine-13*’, ‘*Levine-32*’ and ‘*Samusik*’ were obtained from open-source repositories (Weber and Robinson, 2016) and arc-sinh transformed with a standard cofactor of 5. Doublets, debris and dead cells were removed, and ground-truth labels were taken from the original publications, with manual gating by the respective authors (Levine *et al.*, 2015; Samusik *et al.*, 2016). A 28-colour spectral flow cytometry dataset, ‘*OMIP*’, was obtained from open-source repositories (Mair and Prlic, 2018). Data were arc-sinh transformed with a standard cofactor of 150 and manually gated according to the gating strategy described by the original authors (Mair and Prlic, 2018). The following populations were identified and served as a ground truth for the comparison of results from the clustering algorithms: CD14^+^ monocytes; CD19^+^ CD80^+^ and CD19^+^ CD80^−^ B cells; CD45RA^+^ CCR7^+^ naïve CD3^+^ CD4^+^ T cells; CD45RA^+^ CCR7^+^ naïve CD3^+^ CD8^+^ T cells; CD3^+^ CD4^+^ CD8^+^ double positive (DP) and CD3^+^ CD4^−^ CD8^−^ double negative (DN) T cells; CD56^+^ natural killer (NK) cells; and CD141^+^ dendritic cells (DCs), CD1c^+^ DCs, CD1c^−^ CD141^−^ (DN) DCs and CD123^+^ plasmacytoid dendritic cells (pDCs).

Finally, two in-house generated flow cytometry datasets were used, ‘*Sepsis*’ and ‘*Peritoneal Dialysis*’ (*PD*). Both datasets were acquired using a 16 colour BD LSR Fortessa. *Sepsis* data were derived from nine acute sepsis patients (see Supplementary Methods; Supplementary Table S2), were arc-sinh transformed (standard cofactor of 150) and batch effect corrected using the Harmony algorithm (Burton *et al.*, 2021). Each sample was manually gated for single live CD4^+^ and CD8^+^ T cells, Vδ2^+^ γδ T cells and CD161^+^ Vα7.2^+^ mucosal-associated invariant T (MAIT) cells. The identified lymphocyte populations then served as a ground truth for the comparison of results from the clustering algorithms. PD data were derived from a single adult receiving PD with no previous infections for at least 3 months prior to sampling (Burton *et al.*, 2021). Leukocyte populations in peritoneal effluent were identified as live CD45^+^ immune cells and manually gated for CD3^+^ T cells, CD19^+^ B cells, CD15^−^ CD14^+^ monocytes/macrophages, CD15^+^ neutrophils, CD15^−^ CD14^+/−^ CD1c^+^ DCs and CD15^−^ SIGLEC-8^+^ eosinophils. The identified populations then served as a ground truth for the comparison of results from the clustering algorithms.

Base clustering and graph ensemble methods, and the metrics used to evaluate their performance against ground-truth labels are described in Supplementary Methods (Moon *et al.*, 2019; Pedregosa *et al.*, 2011; Qiu *et al.*, 2011; Van Gassen *et al.*, 2015; Levine et al., 2015; Stassen *et al.*, 2020; Yang *et al.*, 2019). To make analysis manageable, where data exceeded 300 000 observations (which was the case for the *Samusik*, *OMIP*, *Sepsis* and *PD* data), down-sampling was performed. To demonstrate the computational efficiency of GeoWaVe, additional experiments were performed using synthetic data (see Supplementary Methods).

### 2.2Geometric median clustering with weighted voting

Graph ensemble methods address issues of computational complexity by using a heuristic, deriving the consensus from graph representations of the label-association matrix, rather than from the unmanageable co-association matrix. Taking inspiration from this approach, we propose a novel alternative heuristic ensemble clustering method that incorporates information about the original feature space: GeoWaVe, where the clusters generated by base clustering algorithms contributing to an ensemble are summarized by their geometric median. The geometric median (implemented with the *hdmedians* package; Roberts *et al.*, 2017) was chosen over other measures of central tendency because it is robust to outliers, is not necessarily a point from the original data, can handle negative values and is defined in any dimension.

**Fig. 1.**
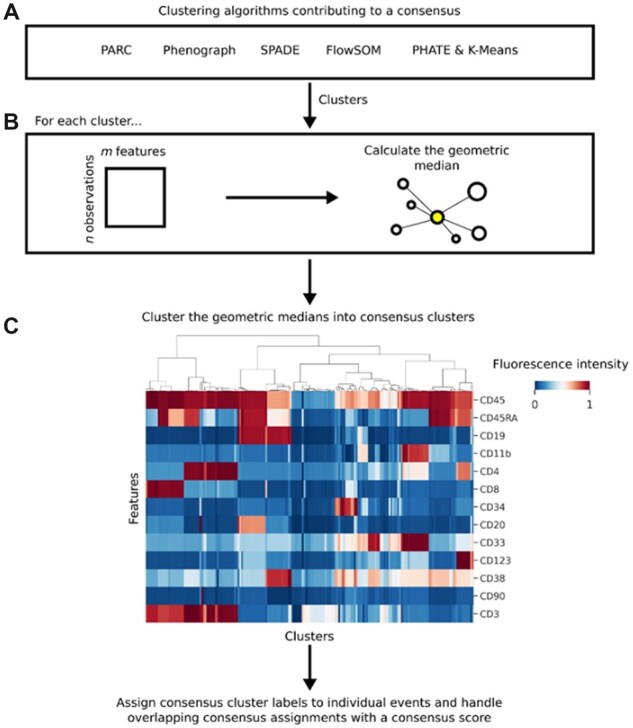
Schematic diagram of the GeoWaVe algorithm. (**A**) Clusters generated by multiple clustering algorithms are pooled, and (**B**) the geometric median for each cluster is calculated to create a matrix of *c* clusters. (**C**) This matrix of cluster geometric medians (clusters of the *Levine-13* data shown here as an example) is clustered into consensus clusters; groups of clusters within similar expression profiles. Consensus cluster labels are then assigned to individual events and overlapping consensus assignments handled with a score that accounts for the distance of the event to the members of each consensus cluster

Using this approach, a summary of the expression profile of all clusters contributing to the consensus is generated, which can subsequently be clustered into consensus clusters (Fig. 1 heatmap); a consensus cluster being a collection of clusters of similar phenotypes. Since each cluster is treated as an individual contribution, differences in the number of clusters provided by each input algorithm are not consequential, meaning GeoWaVe can accept the outputs of any combination of clustering algorithms.

The clusters that contribute to a consensus are overlapping sets, given that each base clustering algorithm is exposed to the same data. Therefore, it is possible that an event can be assigned to more than one consensus cluster. This will occur more frequently for events that sit on the boundary between clusters. To solve this problem, where an event is assigned to multiple consensus clusters a score is calculated for each consensus cluster and the event assigned to the consensus with the maximum score.

Given that a consensus cluster can be defined as a set of clusters *c* ∈ *C*, and a single cluster *c* is a finite set of *n*-dimensional vectors, the geometric median $\hat{u}$ of each cluster *c* can be calculated according to Equation 1 (Roberts *et al.*, 2017):

(1) u^=argmin x ∈ R⁡x-xi2.

For each event *t* assigned to more than one consensus cluster *C*, the Manhattan distance between the event and the geometric median of each member cluster of *C* is computed. The sum of these distances normalized by the size of the consensus $\left|C\right|$ (i.e. the number of clusters within the consensus) gives a weighting factor *p* for the consensus cluster *C* relative to the event *t* (Equation 2):

(2) $$p=\frac{\sum_{c\in C}{\left\|t-\;\hat{u}(c)\right\|}_{1}}{\left|C\right|}.$$

The consensus cluster score for *C* relative to an event *t* is then calculated as the size of the consensus $\left|C\right|$ divided by the weighting factor *p* (Equation 3):

(3) $$\mathrm{score}=\frac{\left|C\right|}{p}.$$

The motivation for the consensus cluster score is derived from the fact that not all clusters are equally defined, and some may be a poor fit for a given event. To account for this possibility, the majority voting algorithm is weighted by the distance from an event to the centre of each cluster that contributes to a consensus. This method ensures that the consensus an event is assigned to is informed by both the number of supporting algorithms (described by the term $\left|C\right|$ in Equation 3) but also the quality of the clusters in that consensus (described by the term *p* in Equation 3).

The choice of clustering algorithm applied to the geometric medians of clusters is ambiguous in that any number of existing methods may be suitable to the task. The advantage of geometric medians as a heuristic is that the expression profile can be visualized easily as a heatmap (Fig. 1), and different clustering methods can be applied and critiqued. This allows the investigator to introduce prior knowledge, such as known phenotypes expected to occur in the data. The ambiguity of the clustering algorithm applied to the geometric median matrix allows for the use of methods such as the ConsensusClusterPlus method (Wilkerson and Hayes, 2010), choosing an optimal number of clusters from a given range. Therefore, an investigator can visualize the geometric medians and choose a range of clusters based on an intuition driven by the biological question.

GeoWaVe is available as part of the CytoCluster package, developed for Python version 3.8 or greater. The CytoCluster package is available on the Python Package Index (PyPI) and offers popular cytometry clustering algorithms, graph ensemble clustering and GeoWaVe ensemble clustering, as well as numerous utilities and plotting tools, delivered through a simple object-orientated application programming interface.

## 3 Results

### 3.1 Graph ensemble clustering methods fail to outperform individual clustering algorithms for cytometry data analysis

Diversity among the members of an ensemble can enhance results (Boongoen and Iam-On, 2018). Ensemble clustering solutions should also take input from informative algorithms suited to the analytical task in question. Therefore, we chose algorithms that have reported good performance for cytometry data analysis, are well understood, have differing underlying methodologies and are computationally efficient.

**Fig. 2.**
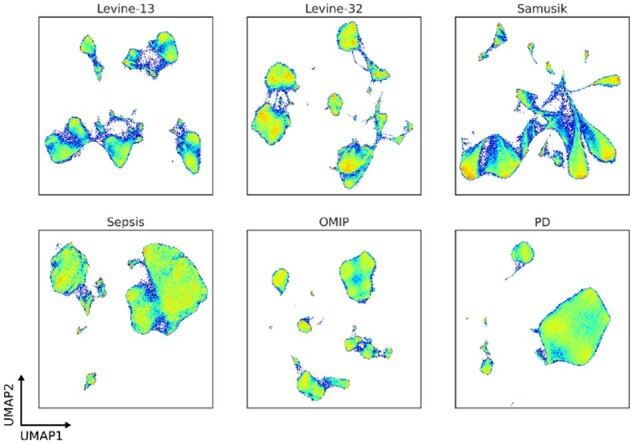
UMAP density plots of the *Levine-13*, *Levine-32*, *Samusik, Sepsis, OMIP* and *Peritoneal Dialysis* (*PD*) data. Colour intensity corresponds to the density of observations in a region of events

We here sought to benchmark ensemble methods from the literature using externally and internally generated data, in particular ensemble methods that scale to large cytometry data (greater than 100 000 data points), namely graph-based methods. Base clustering algorithms and ensemble methods were tasked with clustering three CyTOF datasets with available ground-truth labels. The *Levine-13* data describe a total of 265 627 bone marrow cells from two healthy human donors and include 13 parameters (Supplementary Fig. S1) (Levine *et al.*, 2015). *Levine-32* describes 167 044 bone marrow cells from a single healthy human donor but at higher resolution with 32 CyTOF parameters (Supplementary Fig. S2) (Levine *et al.*, 2015). Examples of challenges presented by these two datasets include overlapping monocyte subsets differentiated by CD11b expression in the *Levine-13* data, and small subsets of B cells differentiated by IgM and IgD expression in the *Levine-32* data. The *Samusik* data describe bone marrow samples with a total of 841 644 cells from 10 C57BL/6J mice and identified 24 populations using 39 CyTOF parameters (Supplementary Fig. S3) (Samusik *et al.*, 2016); the branching topology of which offers a unique challenge to any clustering algorithm aiming to partition data in meaningful ways (Fig. 2).

In addition to these three CyTOF datasets, we included the OMIP-44 28-colour spectral flow cytometry dataset for the identification of human dendritic cell compartments (Mair and Prlic, 2018). Of the 28 parameters, 15 were retained for the identification of the main subsets described by the original authors (Supplementary Fig. S4). To examine the performance on traditional flow cytometry data, two in-house datasets acquired with a 16-colour BD LSR Fortessa were included. The first was for the identification of conventional and non-conventional T cell subsets from peripheral blood mononuclear cells (PBMCs) from patients diagnosed with sepsis (Supplementary Fig. S5A) and the second, for the identification of leukocyte populations in peritoneal effluent from a patient undergoing PD (Supplementary Fig. S5B). Both the *Sepsis* and *PD* data offer unique challenges because of relatively small and ambiguous populations being present amongst a backdrop of more predominant cell types (Fig. 2).

**Fig. 3.**
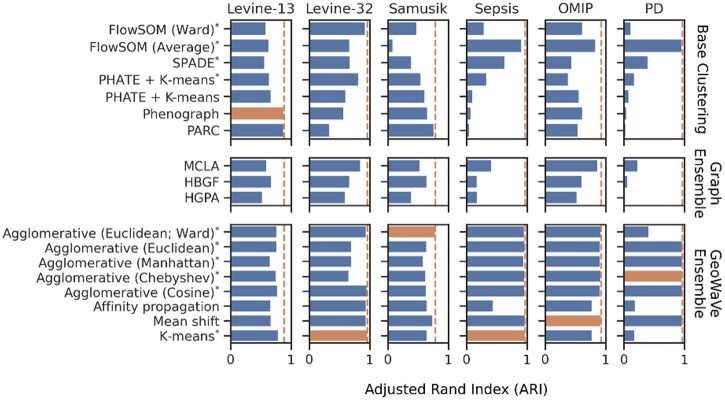
Adjusted rand index (ARI) for base clustering algorithms (top), graph ensemble methods (middle) and GeoWaVe ensemble (bottom) for the six benchmark datasets. The best ARI score for each dataset is shown as a dotted line, and the best performing method for those data is highlighted. *The optimal number of clusters *k* was chosen using the ConsensusClusterPlus method (Wilkerson and Hayes, 2010)

Figure 3 shows the performance of the base clustering algorithms (the algorithms that were used to contribute to ensemble clustering), graph ensemble clustering algorithms and the GeoWaVe variants, measured by adjusted rand index (ARI). In most cases, MCLA offered greater performance compared to the other graph ensemble methods, a finding corroborated by Fowlkes–Mallows index (FMI; Supplementary Fig. S6A) and adjusted mutual information (AMI; Supplementary Fig. S6B). Although in the *Levine-13* and *Levine-32* data graph ensemble methods improved on the performance of algorithms such as SPADE or FlowSOM, in only one of the six datasets (*OMIP*) did any graph ensemble outperform the base clustering algorithms. Based on this evidence, it is difficult to justify the use of graph ensemble methods for cytometry data.

The graph ensemble methods required that the number of consensus clusters *k* be pre-defined. Selection of *k* was performed using internal performance metrics (Supplementary Fig. S7) as described in the Supplementary Methods. To test whether the performance of graph ensemble methods was adversely affected by the chosen method for selecting *k*, the performance of graph-based clustering algorithms was examined across different values of *k* using external evaluation metrics. HBGF was chosen because it had the best runtime of the three graph ensemble methods. Here, performance was optimum for low values of *k* despite the number of ground-truth populations being much larger for the *Levine-13, Samusik* and *OMIP* datasets (Supplementary Fig. S8). The choice of *k* was therefore assumed not to be a factor in the poor performance of graph ensemble methods in this case. Taken together, our findings demonstrate that graph ensemble clustering methods for mass and flow cytometry data performed worse than one or more contributing base clustering solutions.

### 3.2 GeoWaVe outperforms graph ensemble methods and improves upon the performance of base clustering algorithms

To validate GeoWaVe, multiple algorithms for clustering the geometric medians were tried. Affinity propagation and mean shift were compared because of their ability to select the optimal number of clusters from the characteristics of the data. *k*-means and agglomerative hierarchical clustering were also tested, with the optimal number of clusters chosen from a range of clusters using the ConsensusClusterPlus method (Wilkerson and Hayes, 2010). For agglomerative hierarchical clustering, a variety of linkage methods and distance metrics were tried. Agglomerative hierarchical clustering offers an additional advantage to the end use, because consensus clusters can be easily visualized as a dendogram and clustered heatmap, allowing the investigator to choose an appropriate range for the number of consensus clusters driven by their understanding of the underlying biology.

GeoWaVe performance was compared to base clustering algorithms and graph ensemble methods using external evaluation metrics. GeoWaVe outperformed all other methods in five of the six datasets when comparing ARI (Fig. 3) and FMI (Supplementary Fig. S6). GeoWaVe also outperformed graph ensemble methods when comparing ARI, FMI and AMI but failed to outperform base clustering methods in terms of AMI in the *Levine-13* and *Samusik* data.

The effect of the choice of clustering algorithm applied in GeoWaVe was data specific. For the *Levine-13*, *Samusik* and *OMIP* data the choice of the algorithm was negligible, whereas hierarchical clustering for the *Levine-32* data was sensitive to the choice of distance metric. Affinity propagation gave a very poor performance for *Sepsis* data. Likewise, affinity propagation, along with *k*-means and Ward clustering, resulted in poor performance for *PD* data.

### 3.3 GeoWaVe outperforms graph ensemble methods for the detection of under-represented populations

External evaluation metrics used in the prior section offer performance criteria that are independent of the labels, i.e. they do not require a like-to-like matching of cluster and ground-truth labels. Instead, measures of similarity between the cluster labels and ground-truth labels were used. Aghaeepour *et al.* (2013), Samusik *et al.* (2016) and Weber and Robinson (2016) alternatively framed such problems in the context of a classification task: a one-to-one mapping of ground-truth labels to clusters was achieved using the Hungarian algorithm such that the sum of F1 scores across ground-truth labels is maximized, and the precision (positive predictive value), recall (sensitivity) and F1 score (harmonic mean of precision and recall) for each ground-truth label are reported.

**Fig. 4.**
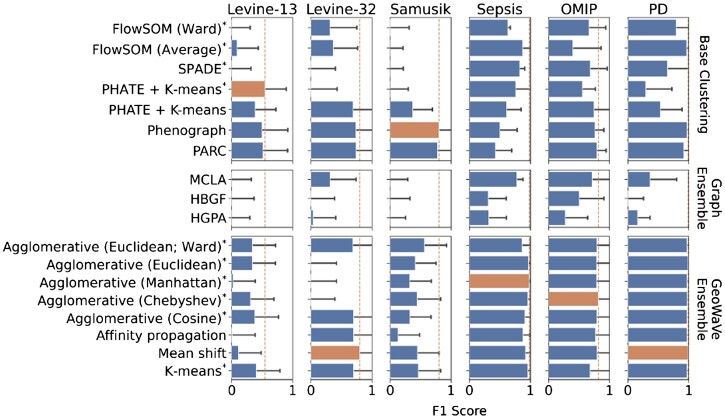
Performance of base clustering algorithms, graph ensembles and GeoWaVe ensembles, after matching cluster labels to ground-truth labels using the Hungarian linear assignment algorithm (as described by Weber and Robinson (2016)) and maximizing the sum of F1 scores across ground-truth label and cluster label pairings. Average F1 scores are reported with error bars showing the standard deviation either side of the average. *The optimal number of clusters *k* was chosen using the ConsensusClusterPlus method (Wilkerson and Hayes, 2010)

This procedure was repeated for the clustering algorithms bench-marked in previous sections and the ensemble clustering solutions. Figure 4 shows the average F1 score for the base clustering algorithms, graph ensemble methods and GeoWaVe along with the standard deviation (error bars) showing the variation in F1 score between populations. The F1 score, precision and recall are reported in Supplementary Figure S9. GeoWaVe continued to outperform graph ensemble methods across the six benchmark datasets but failed to match the F1 score obtained by methods such as PHATE combined with *k*-means in the *Levine-13* data and Phenograph in the *Samusik* data. While MCLA graph ensemble clustering was more comparable to GeoWaVe in the *Sepsis* data when observing F1 score, GeoWaVe clustering still outperformed MCLA in terms of precision, recall and F1 score. GeoWaVe clustering offered optimal average F1 scores for *Levine-13*, *Sepsis*, *OMIP* and *PD* data, and outperformed graph ensemble methods across all datasets.

**Fig. 5.**
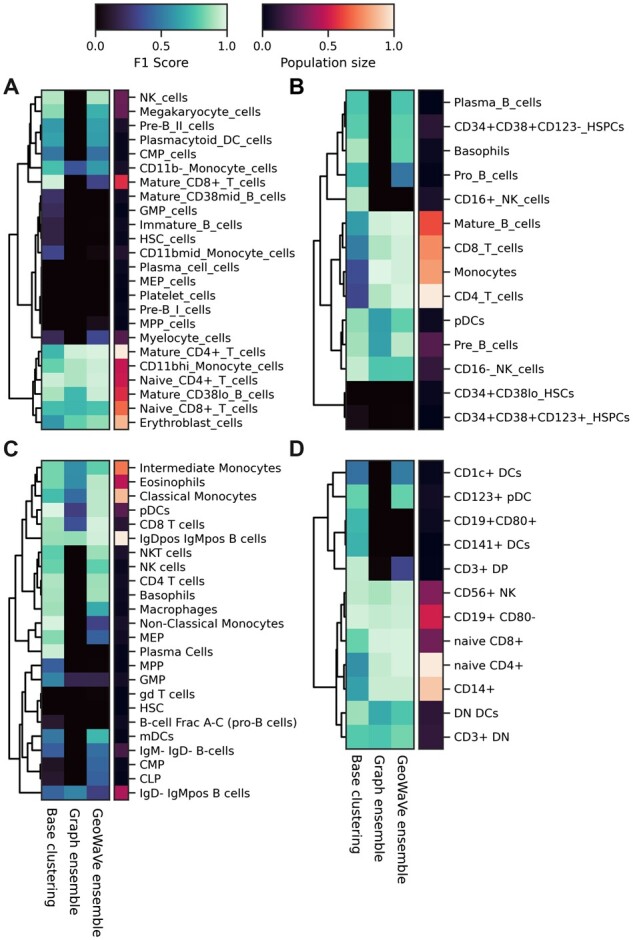
Heatmap of population F1 scores for the *Levine-13* (**A**), *Levine-32* (**B**), *Samusik* (**C**) and *OMIP* (**D**) data. Population level F1 scores are shown for the top-performing algorithm amongst base clustering, graph ensemble and GeoWaVe algorithms. Ground-truth populations (rows) are coloured by F1 score in the central heatmaps, with darker colours indicating a lower F1 score. On the right y-axis, each row is labelled with an additional heatmap that describes the normalized size of the population (total number of events) relative to other populations within the same data

**Fig. 6.**
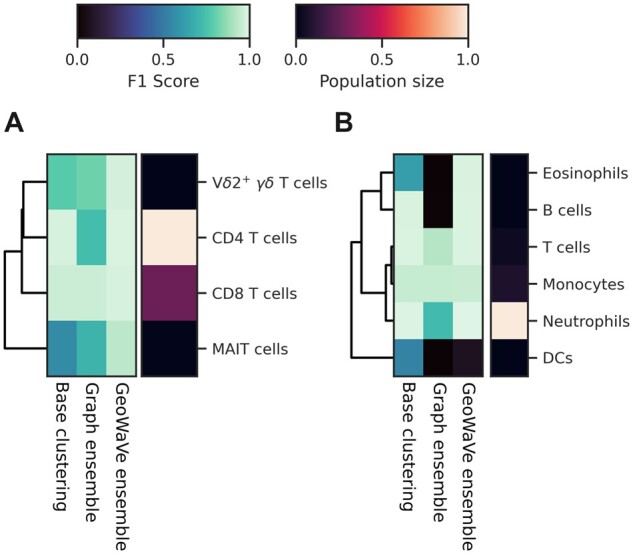
Heatmap of population F1 scores for the *Sepsis* (**A**) and *Peritoneal Dialysis* (*PD*) (**B**) data. Population level F1 scores are shown for the top-performing algorithm amongst base clustering, graph ensemble and GeoWaVe algorithms. Ground-truth populations (rows) are coloured by F1 score in the central heatmaps, with darker colours indicating a lower F1 score. On the right y-axis each row is labelled with an additional heatmap that describes the normalized size of the population (total number of events) relative to other populations within the same data

An advantage to matching clusters to ground-truth populations using the Hungarian algorithm was the ability to compare the performance at the population level. The F1 score for ground-truth populations for the top-performing algorithm from the base clustering, graph ensemble clustering and GeoWaVe ensemble clustering are shown as heatmaps in Figures 5 and 6. Each row includes a measure of the population size as an additional heatmap on the y-axis. The heatmaps demonstrate the superior performance of GeoWaVe compared to graph ensemble methods for the identification of under-represented populations such as pDCs in the *Levine-13* dataset, plasma cells, basophils and pro-B cells in *Levine-32*, pDCs in the *OMIP* data (Fig. 5), B cells and dendritic cells (DCs) in the *PD* data, and MAIT cells in the *Sepsis* data (Fig. 6).

GeoWaVe matched the performance of base clustering algorithms for under-represented cell populations, whereas the graph ensemble clustering algorithms failed to do so. GeoWaVe also showed improved performance over base clustering algorithms for identifying populations such as monocytes, and subsets of T cells in the *Levine-32* data, myeloid DCs (mDCs) in the *Samusik* data, MAIT cells in the *Sepsis* data and eosinophils in the *PD* data. Despite the success of GeoWave in comparison to graph ensemble methods, it still failed to identify some rare subsets completely. In contrast, base-clustering algorithms showed either good performance or identification of at least some of the population. Examples include immature B cells in the *Levine-13* dataset, CD16^+^ NK cells in the *Levine-32* dataset, and plasma cells in the *Samusik* dataset.

### 3.4 GeoWaVe is computationally efficient

Across all variations of the GeoWaVe algorithm run on the six benchmark datasets, the longest recorded runtime was for the 40 parameters Samusik data with 300 000 observations, at a runtime of 2 min and 12 s (Supplementary Tables S3–S5).

**Fig. 7.**
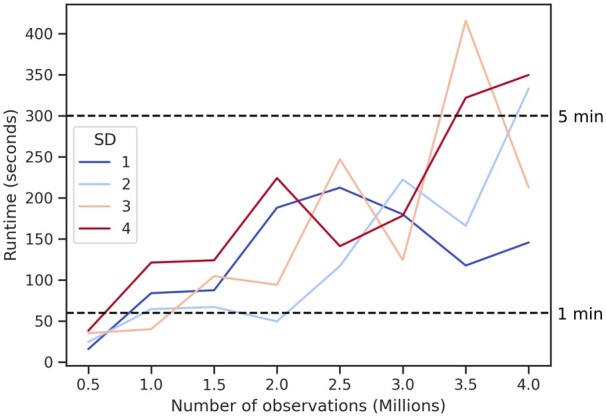
Runtime performance of GeoWaVe algorithm on randomly generated synthetic data consisting of 10 Gaussian data point clouds with an increased number of observations. Four synthetic datasets are shown, each with an increasing standard deviation (SD) used for the generation of Gaussian data point clouds resulting in more overlap between clusters

To assess the ability of GeoWaVe to scale to larger data, we tested it against synthetic data of increasing size and complexity (see Supplementary Methods; Supplementary Fig. S10). The runtime performance of the GeoWaVe algorithm is affected by two attributes of the data: the total number of observations and the overlap between clusters obtained by base clustering algorithms. Increasing overlap between clusters results in more observations being assigned to multiple consensus clusters, and the consensus cluster score (described in Section 2) must be computed for each of these observations. GeoWaVe employs multiprocessing to distribute these calculations across the available cores of a machine, resulting in excellent runtime performance as shown for ten randomly generated Gaussian data point clouds in 15 dimensions (Fig. 7). Using GeoWaVe, we were able to generate ensemble clusters in <10 min, even for datasets scaling to millions of observations. We believe that such runtimes are reasonable and allow investigators to run experiments with a range of hyperparameters.

## 4 Discussion

Cytometry has become a cornerstone of biomedical and healthcare research and is widely used in clinical diagnosis. In many pathological conditions, the understanding of disease mechanisms and how to exploit them for patient benefit relies largely on cytometry, including the diagnosis of conditions like leukaemia and HIV infection, and studying antigen-specific responses in vaccine trials. Historically, cytometry data have been processed and analysed manually. Until recently, this was deemed acceptable given that cytometry instruments could only accommodate relatively few parameters in any experiment. Over the past decade, however, the number of available parameters has increased drastically with the advent of multicolour flow cytometry and mass cytometry, allowing the characterization of even minor populations at the single-cell level and the discovery of novel cell types and new functional features. Traditional approaches no longer suffice—as the number of parameters grows, data analysis is becoming more labour intensive, more subjective and harder to standardize and reproduce across studies and sites. In response to the technological advances, the domain of cytometry bioinformatics is rapidly evolving to provide new computational solutions for data analysis and interpretation such as autonomous gating, supervised classification and unsupervised clustering. Arguably the most impactful technology introduced to this space are clustering algorithms designed specifically for cytometry data analysis, such as SPADE, FlowSOM and Phenograph. The top clustering algorithms alone have already amassed >12k citations in the scientific literature within a few years and are enabling researchers to make rapid progress in their fields—for instance, in the understanding of the immunopathology of COVID-19 that rapidly translated into novel therapies, outcome prediction and vaccine development (Arunachalam *et al.*, 2020; Bolouri *et al.*, 2021; Hadjadj *et al.*, 2020; Mathew *et al.*, 2020).

We here developed GeoWaVe, an ensemble clustering algorithm, as a solution to reduce the variance commonly observed amongst clustering methods in the cytometry literature, where results depend upon hyperparameter choice and the particular context in which they are applied. Presently, there is an absence of a ‘one size fits all’ solution to clustering cytometry data, leaving scientists to rely on exploratory analysis that risks biasing results through data dredging. Ensemble clustering offers an alternative by finding a consensus informed by the results of multiple clustering algorithms exposed to the same data. This multi-view approach theoretically offers robust, consistent and stable solutions (Ghosh and Acharya, 2011; Vega-Pons and Ruiz-Shulcloper, 2011) without biasing the analysis with the assumptions of a single algorithm. The act of employing ensemble clustering also forces the analyst to compare and contrast the results of multiple algorithms, which can be an informative exercise.

Ensemble clustering presents many challenges that come to bear when applied to complex data such as those generated with cytometry. Unlike supervised classification, there are not a defined number of classes provided by labelled examples. Different algorithms may generate different quantities of clusters, which must be compared and consolidated into consensus clusters. Cytometry data also tend to generate large data that can be difficult to handle with conventional computer resources. This is becoming increasingly relevant for studies that intend on phenotyping hundreds or even thousands of subjects.

An existing ensemble approach that can scale to large data and was included in this study is the graph-based methods, such as HGPA, MCLA and HBGF. These techniques were benchmarked against six independent datasets but failed to outperform individual clustering algorithms such as FlowSOM, PhenoGraph or SPADE. In response to this, an alternative heuristic ensemble method named GeoWaVe was developed, which was suitable to the nature of cytometry data. Given that the dimensions of cytometry data are not beyond the comprehension of the investigator and meaningful phenotypes can be determined by considering sets of features, we propose to summarize each cluster contributing to a consensus by its geometric median in the feature space. This can for instance be visualized in a heat map. Our study demonstrates that clustering the matrix of these geometric medians can generate informative consensus clusters.

Our analyses showed that GeoWaVe consistently outperformed HGPA, MCLA and HBGF. The use of geometric medians also provided a useful visual aid when choosing the number of consensus clusters to be formed. By visualizing the heat map of geometric medians in combination with *t*-SNE, UMAP or PHATE embeddings, a suitable number of partitions can easily be estimated. This allows the investigator to introduce informative priors and select clusters based on knowledge of the underlying biology. If uncertain, a range of partitions can be searched using the ConsensusClusterPlus method (Wilkerson and Hayes, 2010). Our approach is novel in its computational efficiency, ability to handle millions of observations and its communication of the consensus clusters to the investigator in a familiar manner that reflects the underlying biology.

The use of geometric medians as a heuristic is not without limitations. Summarizing a cluster using the geometric median tells little of the topology, and a significant loss of information may result in misinformed consensus clusters that are not representative of the data themselves. Additionally, the optimal choice of clustering method applied to the matrix of geometric medians is not immediately apparent and performance can vary depending on the data—for instance, this choice was important to the performance on the *Levine-32, Sepsis* and *PD* data, but less relevant for the *Levine-13*, *Samusik* and *OMIP* data. Of note, the use of a heuristic means that the run-time of GeoWaVe is fast enough to accommodate hyperparameter tuning. The investigator is therefore encouraged to experiment with different clustering algorithms and hyperparameters and inspect the partitions on the geometric median heat maps and embeddings generated from a suitable dimension reduction technique. Although this fails to remove the exploratory approach to clustering of cytometry data, it introduces the multi-view consensus necessary for robust results.

Weber and Robinson (2016) performed a similar assessment of clustering algorithms without the focus on consensus methods and framed their assessment as a classification problem, inspired by the work by Samusik *et al.* (2016). They chose to use F1 score by first mapping clusters to ground-truth labels using the Hungarian algorithm and maximizing F1 scores across reference populations. This methodology was repeated in the present study and supported the conclusion that GeoWaVe ensemble methods outperform the graph ensemble methods of HGPA, MCLA and HBGF. Closer inspection of individual population F1 scores revealed that rare cell populations were often not identified by graph ensemble methods. Although identification of these subsets was improved in GeoWaVe, performance was often worse than individual clustering algorithms and some populations, such as platelets in the *Levine-13* data, remained unidentified. The performance of the base clustering algorithms for many rare cell populations was also poor, possibly impacting the performance of ensemble outputs. Further work is needed to generate clustering methodologies that directly address this limitation.

There is a significant flaw in the assessment of clustering performance through F1 score. Mapping clusters to ground-truth labels in such a way implies that a one-to-one relationship must exist between the clusters generated and the reference populations. Clustering analysis can be complicated by sub-structures in data captured as clusters but absent in the ground-truth labels. If the purpose of clustering cytometry data is to identify a precise number of clusters, then this form of evaluation seems justified although one could argue that in such a scenario a supervised classification approach might be more suitable. Clustering analysis tends to be applied in the interest of discovery when the number of clusters is unknown. Despite this flaw, it was deemed necessary to replicate the methods of Weber and Robinson (2016), which was informative of the role population size plays. It showed that although the consensus clustering of geometric medians outperforms graph-based methods, there is still work to be done to ensure rare cell populations do not go undetected with this technique. It would be advisable that if rare cell populations are suspected to be present, that the consensus is formed by methods with high resolution such as those formed on nearest-neighbour graphs (Levine *et al.*, 2015; Samusik *et al.*, 2016; Stassen *et al.*, 2020).

Future work should focus on more diverse ensemble clustering. In this work, four classes of algorithm were chosen based on their popularity in the cytometry literature and their available implementations. However, there is a wide variety of further clustering algorithms that could be explored for inclusion in ensemble clustering. There are ongoing efforts to address the computational complexity, such as improvements to SC3. Other solutions to the computational complexity may come from advances in the statistical and computational literature, such as consensus formed on heuristics of cluster similarity using metrics such as the Jaccard index (Khedairia and Khadir, 2022). In the meantime, clustering on geometric medians is likely to be a viable solution for cytometry data analysis. We are confident that the availability of user-friendly but powerful ensemble clustering methods has the potential to represent a major advance in big data analysis, with implications for an improved understanding of cellular responses, clinical conditions, and therapeutic and diagnostic options.

## Supplementary Material

btac751_Supplementary_Data

## Data Availability

The *Levine-13*, *Levine-32* and *Samusik* datasets are available from Flow Repository, repository number FR-FCM-ZZPH (Weber and Robinson, 2016). The *OMIP-44* data are available from Flow Repository under the repository number FR-FCM-Z32U. The *Sepsis* and *PD* data underlying this article are available at https://doi.org/10.5281/zenodo.7134723.
