# Supplementary material for: GeoWaVe: geometric median clustering with weighted voting for ensemble clustering of cytometry data
Source: Bioinformatics. 2022 Nov 22;39(1):btac751. doi: 10.1093/bioinformatics/btac751 (PMC9805571; doi:10.1093/bioinformatics/btac751)
Supplement: btac751_Supplementary_Data [file btac751_supplementary_data.pdf]

SUPPLEMENTARY DATA

Geometric median clustering with weighted voting (GeoWaVe): A novel consensus method for ensemble clustering of cytometry data

Ross J. Burton[1,2,*], Simone M. Cuff[1], Matt P. Morgan[2],
Andreas Artemiou[3,†], Matthias Eberl[1,4,†]

[1]Division of Infection and Immunity, School of Medicine, Cardiff University, Cardiff CF14 4XN, United Kingdom; [2]Adult Critical Care, University Hospital of Wales, Cardiff and Vale University Health Board, Cardiff CF14 4XW, United Kingdom; [3]School of Mathematics, Cardiff University, Cardiff CF24 4AG, United Kingdom; [4]Systems Immunity Research Institute, Cardiff University, Cardiff CF14 4XN, United Kingdom

[*] To whom correspondence should be addressed

[†] The authors wish it to be known that, in their opinion, the last two authors should be regarded as Joint Senior Authors.

1. Supplementary methods

1.1 Sepsis data

Ethics statement. Recruitment of sepsis patients was approved by the Health and Care Research Wales Research Ethics Committee under reference 17/WA/0253, protocol number SPON1609-17 and IRAS project ID 231993 ("Innate-like T cells in sepsis [ILTIS]: Implications for early diagnosis and rescue of immunosuppression") and conducted according to the principles expressed in the Declaration of Helsinki. All participants provided written informed consent for the collection of samples and their subsequent analysis. A waiver of consent system was used when patients were unable to provide prospective informed consent due to the nature of their critical illness or therapeutic sedation at the time of recruitment. In all cases, retrospective informed consent was sought as soon as the patient recovered and regained capacity. In cases where a patient passed away before regaining capacity, the initial consultee's approval would stand.

Subjects. Sepsis patients were over 18 years old with a positive diagnosis according to the Third International Consensus Definitions for Sepsis and Septic Shock ('Sepsis-3'). They were cared for in the intensive care unit at the University Hospital of Wales in Cardiff, United Kingdom, and were recruited within 36 hours of the presumed onset of the infective illness when they already had or would require arterial cannulation as part of standard treatment. Patients were excluded if they were pregnant or breastfeeding, or were females of childbearing age in whom a pregnancy test had not been performed; if they had severe immune deficiency, for example a diagnosis of AIDS or treatment with anti-rejection transplant drugs or high dose corticosteroids; if they had haematologic malignancy or ongoing chemotherapy; if they had severe liver failure (Child's score III or worse); if they were adjudged by the admitting clinician to be unlikely to survive for the duration of the study period regardless of treatment; if they

were admitted post-cardiac arrest; or if they had an underlying impairment of higher function that would make it impossible for informed consent to be given upon recovery (*e.g.* severe learning disability). This study cohort comprised a total of *n*=9 sepsis patients, with an age ranging from 18-83 years (median 71 years), 40% of which were female.

Flow cytometry. Peripheral blood mononuclear cells were stained after Ficoll-Paque PLUS (Fisher Scientific) separation of blood, using monoclonal antibodies against CD3, CD4, CD8, CD161, TCR-Vα7.2, TCR-Vδ2, TCR-pan-γδ, CD45RA, CCR7 and CD27 (see **Supplementary Table S2**). Cells were acquired on a 16-colour BD LSR Fortessa flow cytometer (BD Biosciences). Live single cells were gated based on side and forward scatter area/height and exclusion of live/dead staining (fixable Aqua; Invitrogen). All data were pre-processed with FlowAI version 3.14 (Bioconductor) to remove poorly acquired events and outliers.

1.2 Base clustering methods

The following base clustering algorithms were considered individually, and their outputs served as input to ensemble clustering: FlowSOM ("self-organising map") (Van Gassen *et al.*, 2015), PHATE ("potential of heat-diffusion for affinity-based trajectory embedding") (Moon *et al.,* 2019) with *k*-means clustering, SPADE ("spanning-tree progression analysis of density-normalized events") (Qiu *et al.,* 2011), Phenograph (Levine *et al.,* 2015) and PARC ("phenotyping by accelerated refined community-partitioning") (Stassen *et al.* 2020). These algorithms are computationally efficient and have shown good performance for cytometry data analysis. For each base clustering algorithm, experimentation with multiple input parameters was performed to give the best possible performance. The number of clusters generated for each method was determined either as a property of the clustering method (as is the case with Phenograph and PARC), selected from a suitable range using the popular

ConsensusClusterPlus method (Wilkerson and Hayes, 2010), or a suitable fixed value was chosen. The choice of the desired number of clusters, either as a range of values or a fixed value, would be driven by an existing biological understanding of the data in general use. For all benchmark data, a range of 5 to 30 was chosen to capture a large range of possible clusters. In the case of PHATE combined with k-Means, clustering was performed with k selected using ConsensusClusterPlus and then performed again with a fixed k of 20, a decision made to increase the diversity of input clusters to the ensemble algorithms.

1.3 Graph-based ensemble clustering

The output of the base clustering algorithms was used to generate a label-association matrix (m clusters × n observations) which served as input for MCLA (meta-clustering algorithm), HGPA (hyper-graph partitioning algorithm) and HBGF (hybrid bipartite graph formulation), three graph ensemble clustering algorithms that have been successfully applied to scRNA-seq data (Yang *et al.,* 2018). The graph ensemble clustering algorithms were implemented using the ClusterEnsembles Python package (https://github.com/827916600/ClusterEnsembles).

For graph ensembles, a required hyperparameter is the number of final partitions in the consensus solution. This problem was addressed in the base clustering algorithms by searching a range of possible clusters and using ConsensusClusterPlus. This approach required sub-sampling the feature space and computing the co-association matrix for each value of k (the number of clusters). The cumulative distribution function (CDF) for each co-association matrix was generated, and the optimal k chosen where the CDF is maximum. This was applicable to methods such as FlowSOM and SPADE that use a heuristic or down-sampled feature space but was intractable for graph-based consensus clustering techniques that construct graph representations of a $m \times n$ label-association matrix. Therefore, the optimal number of consensus partitions was chosen using internal metrics (metrics that use internal information from the

clustering process to evaluate the quality of a clustering, *e.g.* the variation within clusters or the degree of overlap between clusters). Ensemble clustering was repeated over a range of *k*; chosen as the smallest and largest number of clusters amongst base clustering algorithms. Four internal metrics, implemented in Scikit-Learn (Pedregosa *et al.*, 2011), were chosen for their ease of interpretation:

- **Calinski-Harabasz score** is calculated as the ratio of the sum of between-cluster dispersion and the sum of within-cluster dispersion. Higher values correspond to better defined clusters.
- **Davies-Bouldin index** compares each cluster to every other cluster measuring similarity as the ratio of within-cluster distances to between-cluster distances. Lower values indicate better define clusters.
- **Distortion score** provides a measure of the compactness of clusters, measured as the average squared distance between each point in a cluster and the cluster centroid.
- **Silhouette coefficient** is measured for each observation and calculated as the distance between the observation and nearest cluster the observation is not a member of (a), minus the mean intra-sample distance (b; the distance between the observation and all other observations in the same cluster), divided by the maximum of a and b. Values are reported between 1 and –1, with values near to 0 indicating overlapping clusters, and negative values generally indicating that an observation was assigned to the wrong cluster.

Internal metrics were measured for 1000 events with 100 resamples and the distributions were plotted for each *k*. The optimal *k* for graph ensemble clustering was chosen as the value that presented the best performance across all internal metrics.

1.4 External metrics for evaluating clustering performance

The performance of clustering algorithms in comparison to ensemble methods was evaluated using external metrics that compare cluster results to ground-truth labels (Pedregosa *et al.*, 2011). The following external metrics were chosen:

- **Adjusted Rand Index (ARI)** is a symmetric measure that considers all pairs of samples and counts those assigned to the same or different clusters in the predicted and ground truth labels. The adjusted rand index is corrected-for-chance by comparison to the expected value from a permutation model.
- **Fowlkes-Mallows Index (FMI)** can be calculated by forming a contingency table of common objects between clusters and the ground truth labels, and captures the geometric mean between precision and recall, therefore having a similar interpretation to the F1 score.
- **Adjusted Mutual Information (AMI)** is the mutual information adjusted for chance. Mutual information is derived from information theory and aims to quantify the amount of shared information between the predicted clusters and the ground-truth populations. Mutual Information is not adjusted for chance and will tend to increase as the number of clusters increases, regardless of the quality of additional clusters. To remedy this, AMI first calculates the expected value for mutual information and adjusts for chance in a similar form to the Adjusted Rand Index. The AMI scores clustering results between 0 and 1, where random label assignments would give a score of 0, but perfect clustering would have a score of 1.

1.5 GeoWaVe runtime experiments with synthetic data

Randomly generated synthetic data were used to test the ability of GeoWaVe to scale to larger data. Synthetic data were generated using the *make_blobs* function from the Scikit-Learn library (Pedregosa *et al.*, 2011). Data were generated with 15 dimensions (features), ranging

from 500,000 to 4,000,000 observations in four batches, with increasing cluster standard deviations from one to four. In total, 32 datasets were generated each containing ten Gaussian clusters.

The synthetic datasets were clustered using three separate *k*-means algorithms, each with a different random seed and number of expected clusters (8, 10 and 12, respectively). Mini-batch processing with a batch size of 1024 was used to scale the *k*-means clustering to large data. The outputs of the *k*-means clustering algorithms served as input to a GeoWaVe clustering algorithm using Euclidean Ward hierarchical clustering of geometric medians.

2. Supplementary Tables

Dataset	Number of observations	Number of parameters (of which utilised for clustering)	Technology	Repository	Reference
Levine-13	167,044	13	Mass Cytometry (CyTOF)	Flow Repository no. FR-FCM-ZZPH	Weber and Robinson, 2016
Levine-32	265,627	32	Mass Cytometry (CyTOF)	Flow Repository no. FR-FCM-ZZPH	Weber and Robinson, 2016
Samusik	841,644*	40	Mass Cytometry (CyTOF)	Flow Repository no. FR-FCM-ZZPH	Weber and Robinson, 2016
OMIP	2,805,957*	28 (15)	Spectral Flow Cytometry	Flow Repository no. FR-FCM-Z32U	Mair and Prlic, 2018
Sepsis	362,361*	10 (6)	Flow Cytometry	Zenodo; doi:10.5281/zenodo.7134723	Unpublished
Peritoneal Dialysis (PD)	6,333,084*	11 (9)	Flow Cytometry	Zenodo; doi:10.5281/zenodo.7134723	Burton *et al.*, 2021

Supplementary Table S1. Description of the benchmarking datasets employed for assessment of ensemble clustering algorithms.

* Data were down-sampled before analysis.

Marker	Fluorochrome	Clone	Isotype	Company
CD3	APC/FIRE	SK7	Mouse IgG1, k	Biolegend
CD4	PE-Cy5.5	S3.5	Mouse IgG2α, κ	Life Technologies
CD8α	BV711	RPA-T8	Mouse IgG1, κ	Biolegend
CD14	V500	M5E2	Mouse IgG2α, κ	BD Biosciences
CD16	FITC	3G8	Mouse IgG1, κ	BD Biosciences
CD19	V500	HIB19	Mouse IgG1, κ	BD Biosciences
CD27	PE-Cy7	M-T271	Mouse IgG1, κ	Biolegend
CD45RA	PE Dazzle	HI100	Mouse IgG2b, κ	Biolegend
CD57	FITC	NK-1	Mouse IgM, κ	BD Biosciences
CD161	APC	191B8	Mouse IgG2α, κ	Miltenyi
CD197 (CCR7)	BV421	G043H7	Mouse IgG2α, κ	Biolegend
TCR-pan-γδ	PE-Cy5	IMMU510	Mouse IgG1, κ	Beckman Coulter
TCR-Vα7.2	BV605	3C10	Mouse IgG1, κ	Biolegend
TCR-Vδ2	PE	B6	Mouse IgG1, κ	BD Biosciences

Supplementary Table S2: Flow cytometry staining panel for the analysis of T cells in PBMCs from sepsis patients.

						Agglomerative hierarchical clustering				
**Dataset**	**No. of observations**	**No. of parameters**	***k*-Means**	**Mean shift**	**Affinity Propagation**	**Ward**	**Manhattan**	**Euclidean**	**Cosine**	**Chebyshev**
Levine-13	167,044	13	84.9 s	43.6 s	33.2 s	57.7 s	30.1 s	52.7 s	42.8 s	47.3 s
Levine-32	265,627	32	47.2 s	58.6 s	55.3 s	53.6 s	31.7 s	47.8 s	43.3 s	47.4 s
Samusik	300,000	40	2 min 12 s	1 min 55 s	1 min 56 s	1 min 57 s	1 min 25 s	1 min 55 s	1min 54 s	1 min 43 s
OMIP	300,000	15	47.3 s	37.6 s	48.2 s	44.2 s	25.9 s	25.7 s	15.9 s	26.7 s
Sepsis	300,000	6	13.1 s	10.5 s	18.3 s	14.3 s	13.4 s	12.4 s	14.6 s	14.6 s
PD	300,000	9	1 min 22 s	8.2 s	1 min 13 s	1 min 36 s	6.2 s	6.39 s	8.9 s	9.2 s

Supplementary Table S3. Runtime performance of GeoWaVe ensemble clustering algorithms on benchmark data. All algorithms were run on an Ubuntu 20.04 operating system with an Intel i7-12700K processor with 12 cores and 32 gigabytes of RAM.

Dataset	No. of observations	No. of parameters	MCLA	HGPA	HBGF
Levine-13	167,044	13	35.3 s	14.7 s	14.7 s
Levine-32	265,627	32	55.8 s	22.6 s	22.6 s
Samusik	300,000	40	11.9 s	25.5 s	25.5 s
OMIP	300,000	15	14 s	25.7 s	25.7 s
Sepsis	300,000	6	15.6 s	25.8 s	25.8 s
PD	300,000	9	8.6 s	25.4 s	25.4 s

Supplementary Table S4. Runtime performance of graph ensemble clustering algorithms on benchmark data. All algorithms were run on an Ubuntu 20.04 operating system with an Intel i7-12700K processor with 12 cores and 32 gigabytes of RAM. MCLA, meta-clustering algorithm; HGPA, hyper-graph partitioning algorithm; HBGF, hybrid bipartite graph formulation.

Dataset	No. of observations	No. of parameters	FlowSOM	Phenograph	PARC	SPADE	PHATE + *k*-Means	PHATE + *k*-Means*
Levine-13	167,044	13	2.7 s	3 min 47 s	1 min 17 s	11 min 19 s	24.7 s	12 min 3 s
Levine-32	265,627	32	5.1 s	26 min 40 s	1 min 11 s	20 min 4 s	1 min 18 s	12 min 6 s
Samusik	300,000	40	5.8 s	34 min 4 s	1 min 35 s	13 min 8 s	1 min 9 s	12 min 6 s
OMIP	300,000	15	5.4 s	6 min 12 s	2 min 53 s	7 min 20 s	29.1 s	10 min 20 s
Sepsis	300,000	6	2.8 s	4 min 40 s	2 min 22 s	18 min 24 s	27.5 s	11 min 27 s
PD	300,000	9	2.9 s	7 min 13 s	1 min 17 s	1 min 17 s	2 min 57 s	10 min 46 s

Supplementary Table S5. Runtime performance of base clustering algorithms on benchmark data. All algorithms were run on an Ubuntu 20.04 operating system with an Intel i7-12700K processor with 12 cores and 32 gigabytes of RAM.

* the optimal number of clusters *k* was chosen using the ConsensusClusterPlus method (Wilkerson and Hayes, 2010).

3. Supplementary Figures

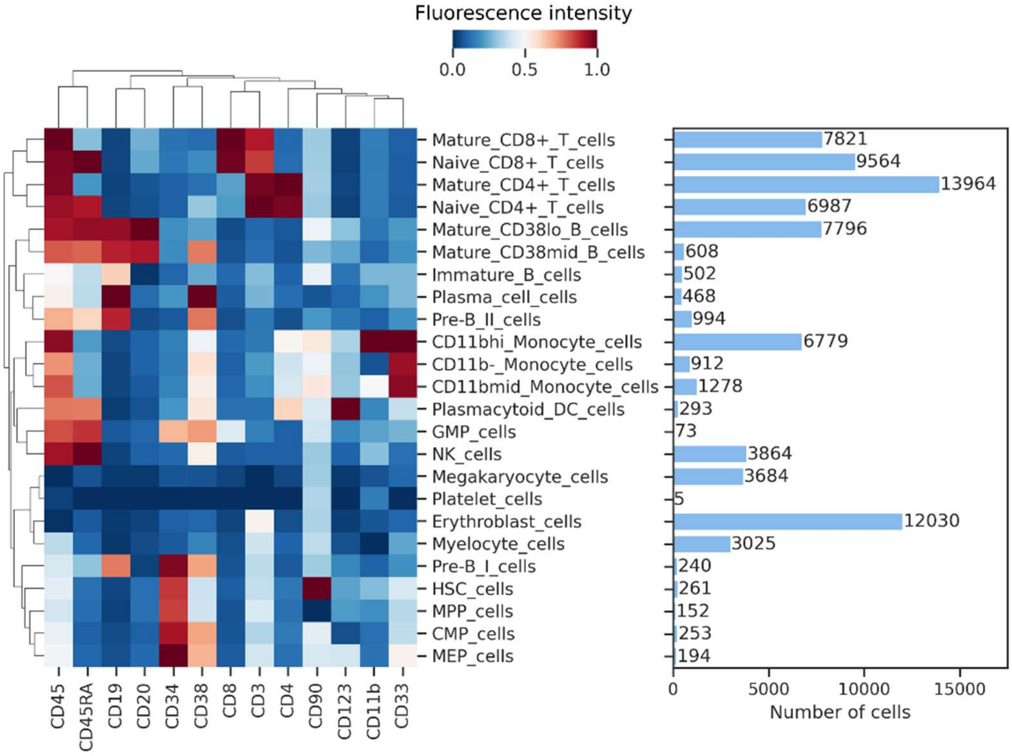

Supplementary Figure S1: Expression profile of the 13-parameter *Levine-13* CyTOF data and the total number of observations for each ground-truth population. The heatmap shows the expression intensity of the cell surface markers indicated, normalised to a range between 0 and 1.

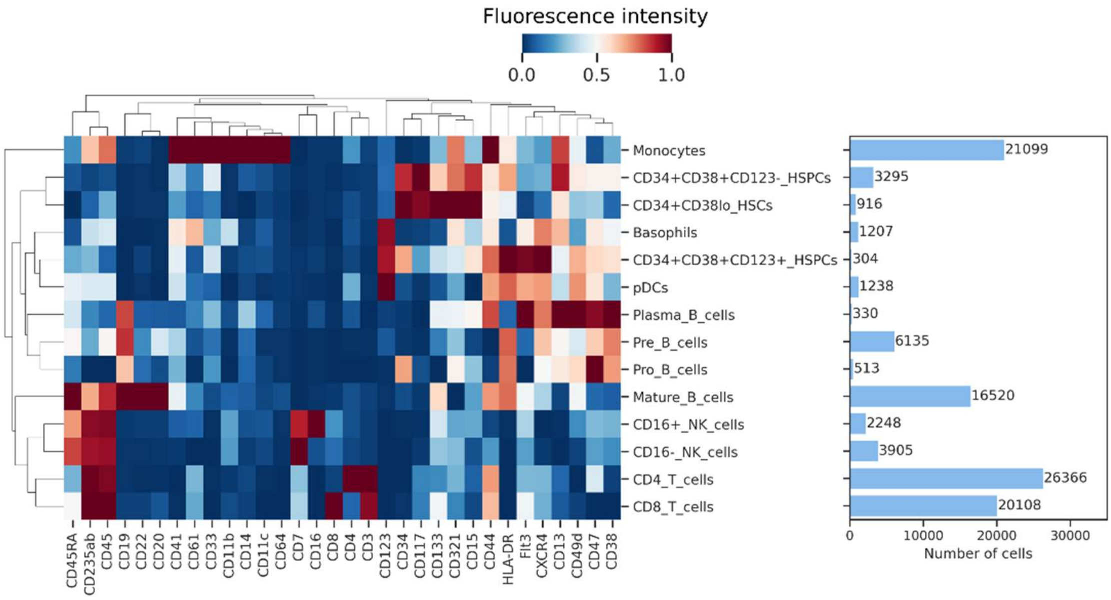

Supplementary Figure S2: Expression profile of the 32-parameter *Levine-32* CyTOF data and the total number of observations for each ground-truth population. The heatmap shows the expression intensity of the cell surface markers indicated, normalised to a range between 0 and 1.

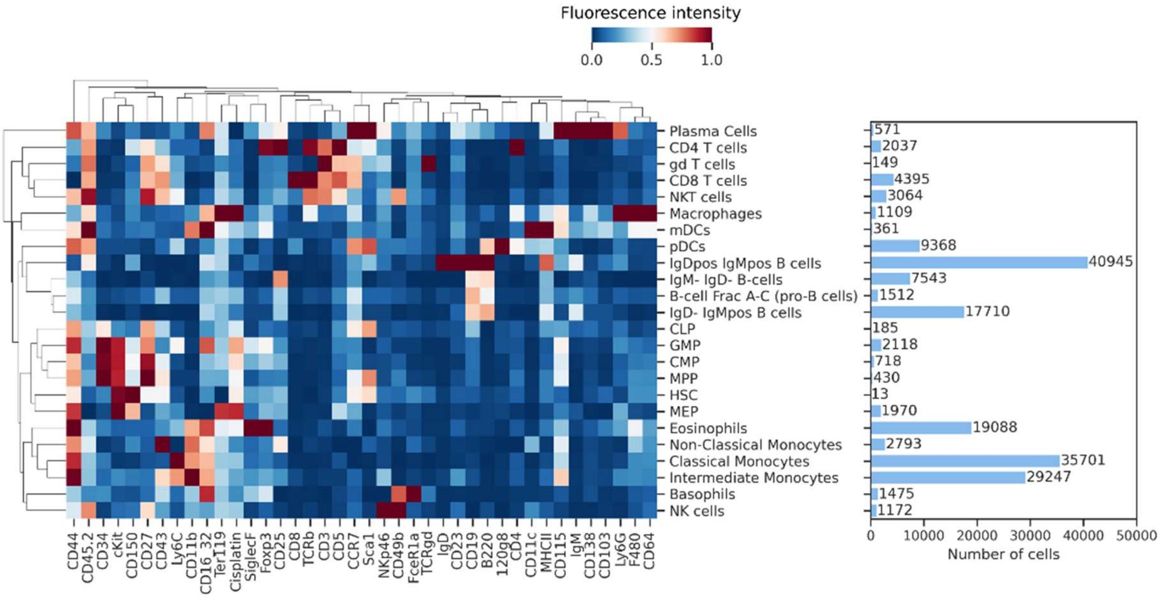

Supplementary Figure S3: Expression profile of the 39-parameter *Samusik* CyTOF data and the total number of observations for each ground-truth population. The heatmap shows the expression intensity of the cell surface markers indicated, normalised to a range between 0 and 1.

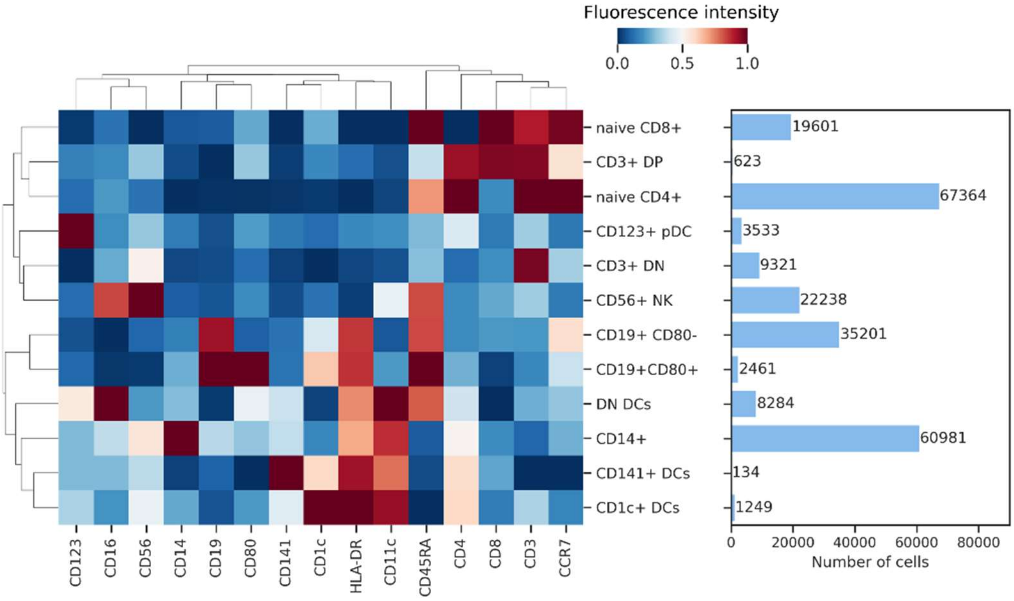

Supplementary Figure S4: Expression profile of lineage markers amongst T cell clusters within the 12-parameter *Sepsis* flow cytometry data and the total number of observations for each ground-truth population. The heatmap shows the expression intensity of the cell surface markers indicated, normalised to a range between 0 and 1.

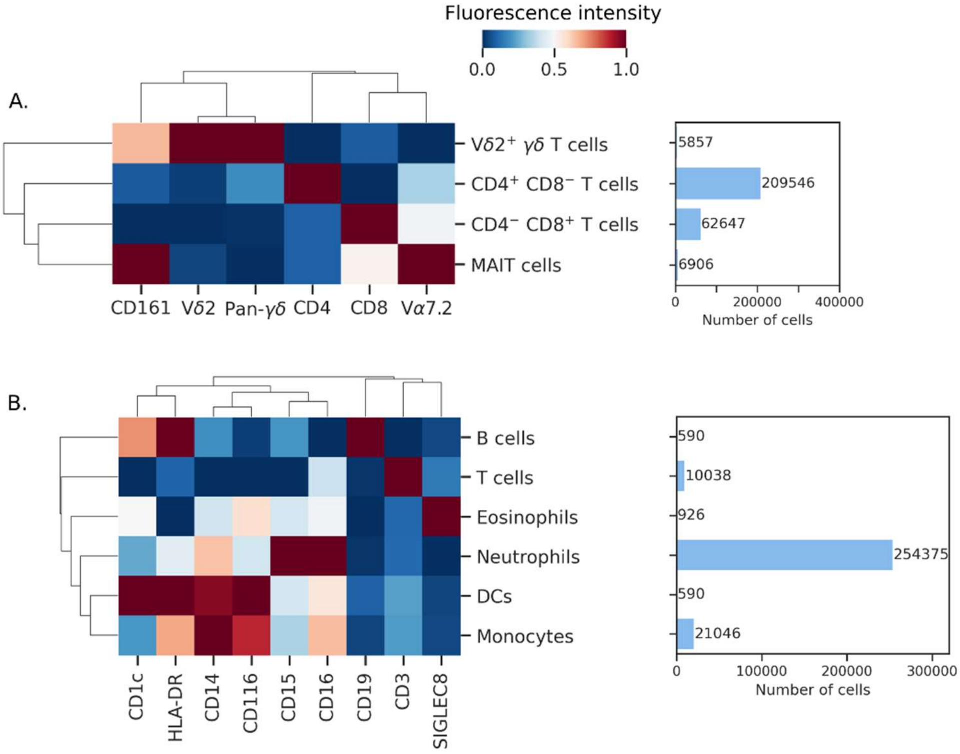

Supplementary Figure S5: Expression profile of lineage markers amongst T cell clusters within from *Sepsis* flow cytometry data (A) and expression profile amongst leukocyte subsets isolated from peritoneal effluent taken from a patient undergoing *Peritoneal Dialysis* (B). The total number of observations for each ground-truth population are shown as a bar chart. The heatmap shows the expression intensity of the cell surface markers indicated, normalised to a range between 0 and 1.

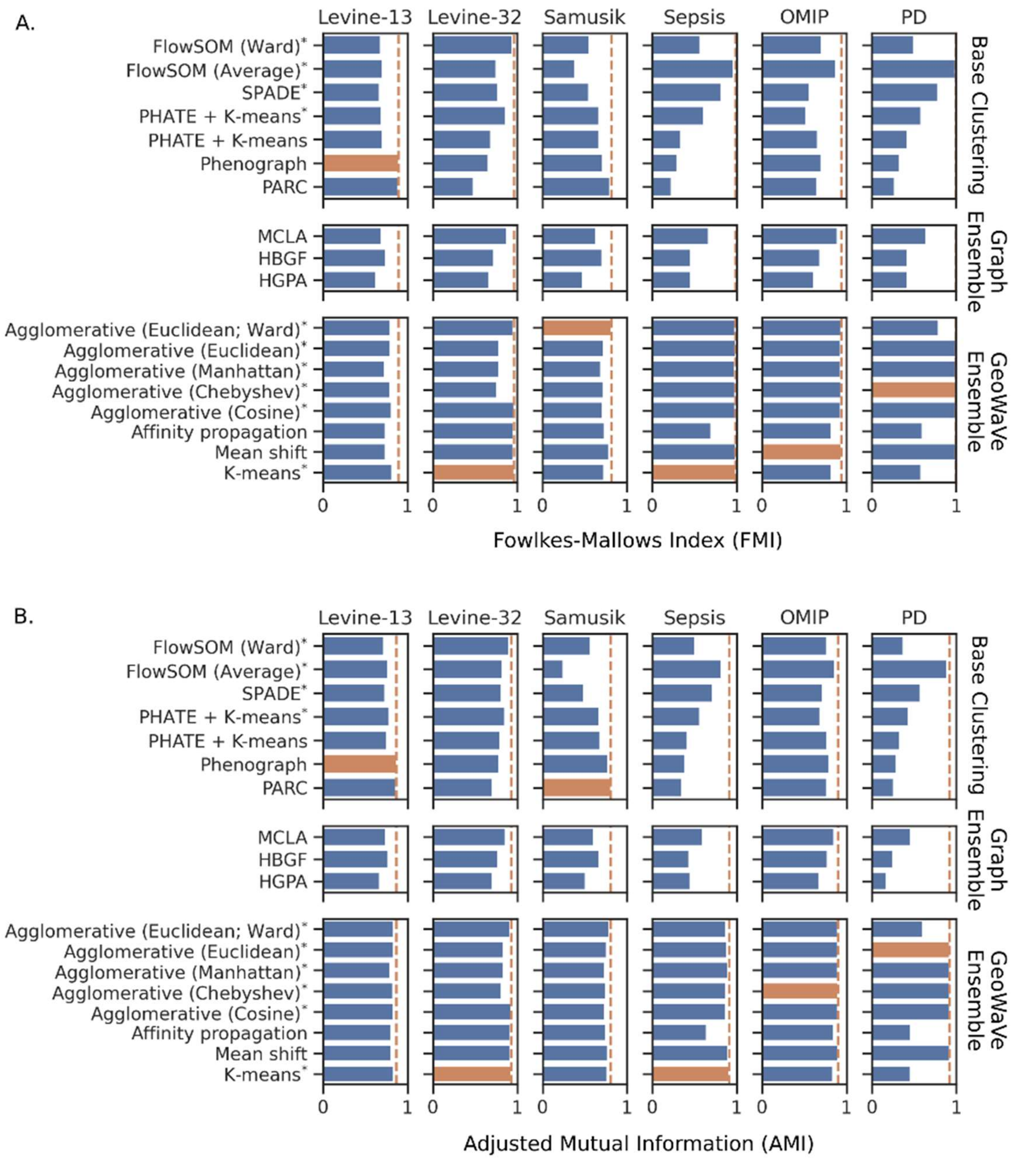

Supplementary Figure S6: Fowlkes-Mallows index (FMI) (A) and Adjusted Mutual Information (AMI) (B) for base clustering algorithms (top), graph ensemble methods (middle) and GeoWaVe ensemble (bottom) for the six benchmark datasets. The best FMI and AMI score for each dataset are shown as a dotted orange line, and the best performing method for those data are coloured in orange.

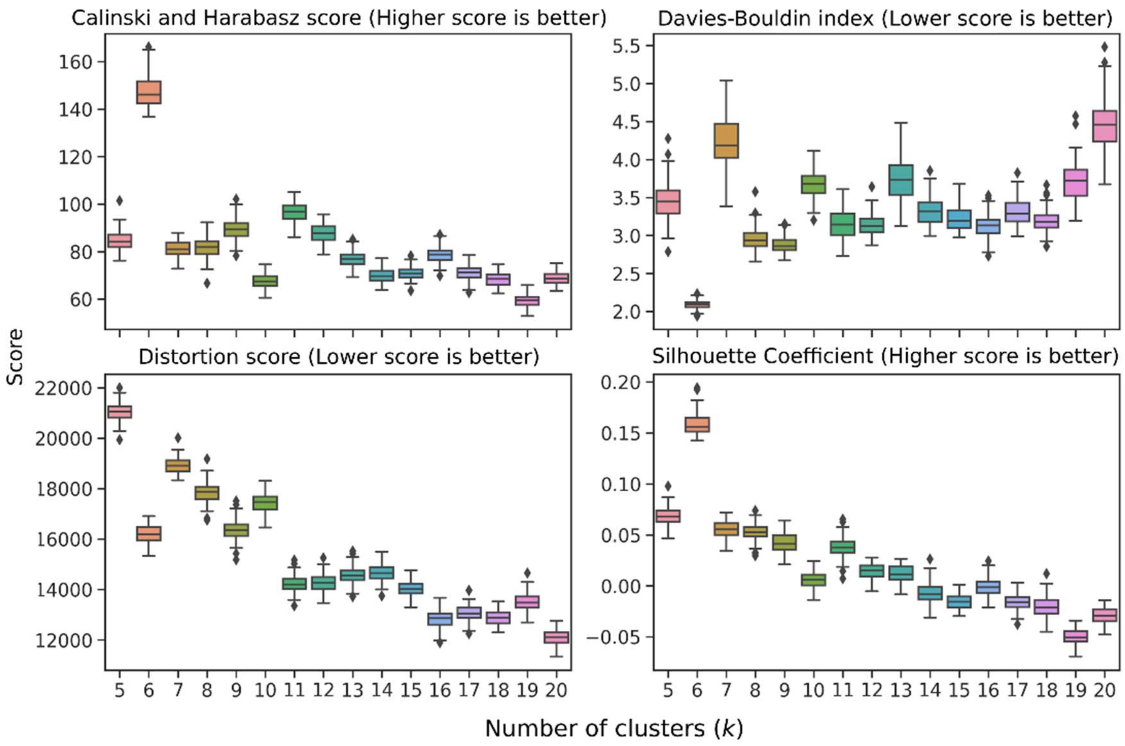

Supplementary Figure S7: Internal metrics for a range of final consensus clusters (*k*) as generated by HGPA clustering of *Levine-13* data. The optimal *k* (chosen as *k*=6 in the example shown) is visually determined as the value where Calinski-Harabasz score and Silhouette coefficient are maximised, whilst Davis-Bouldin index and distortion score are minimised.

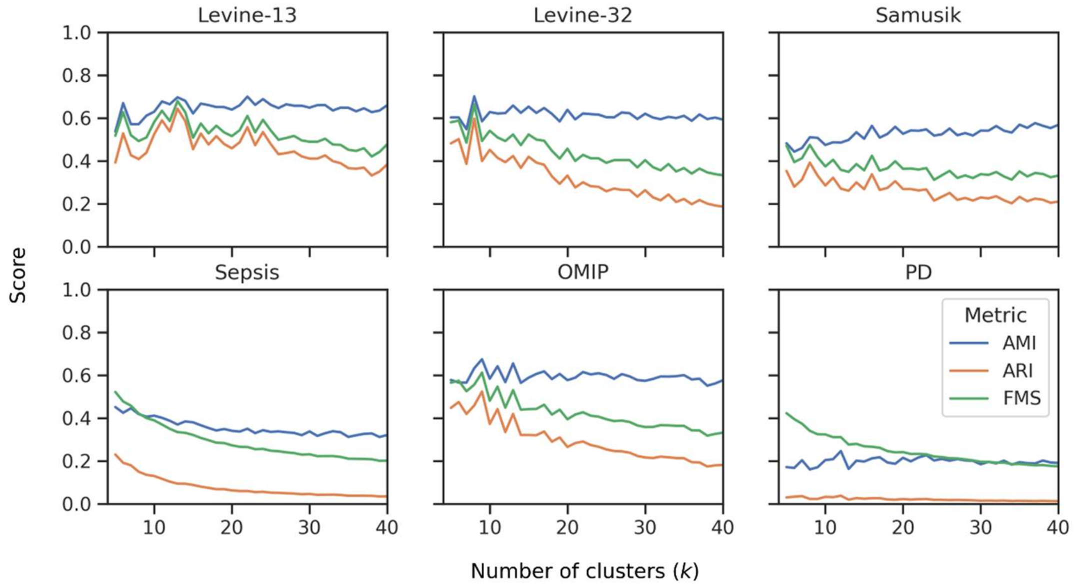

Supplementary Figure S8: Adjusted rand index (ARI), adjusted mutual information (AMI) and Fowlkes-Mallows index (FMI) when the number of consensus clusters (*k*) is varied for HBGF ensemble clustering.

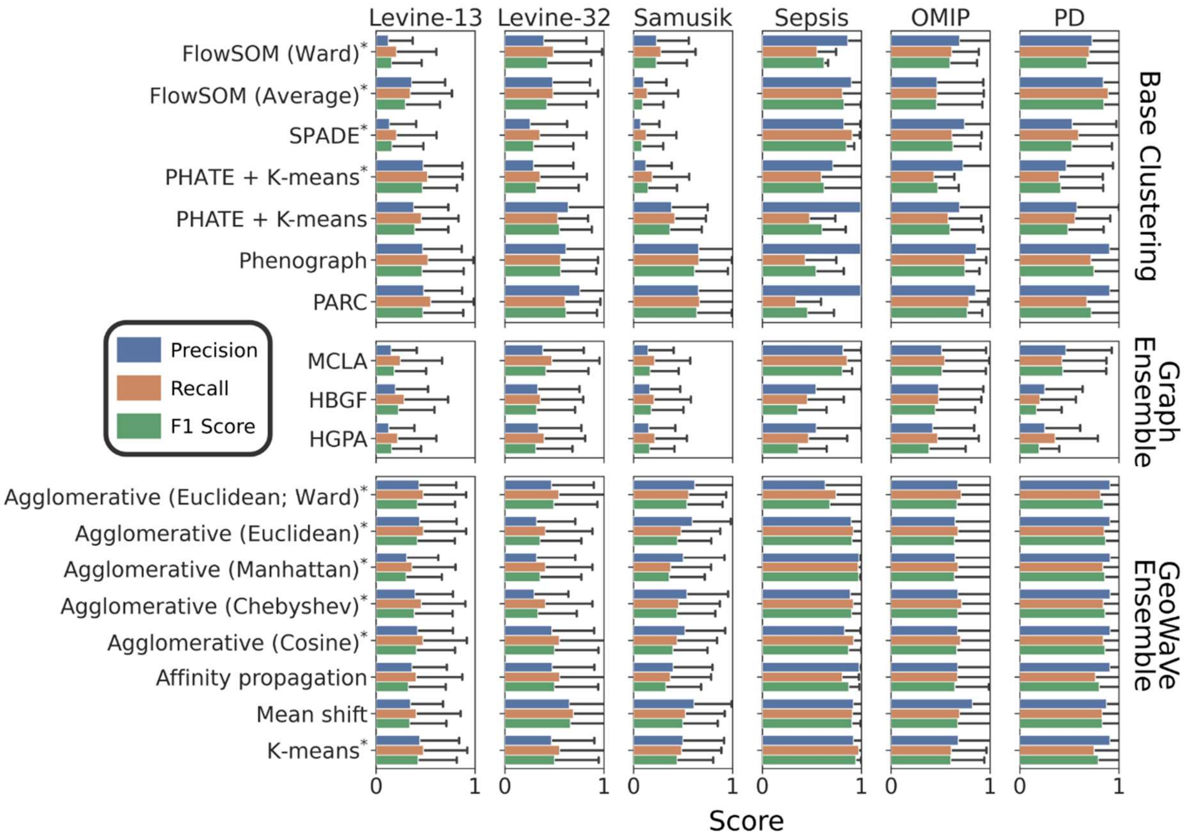

Supplementary Figure S9: Average precision (blue), recall (orange), and F1 score (green) and their standard deviation (error bars) for base clustering (top), graph ensemble (middle), and GeoWaVe ensemble algorithms (bottom) across the six benchmarking datasets. * the optimal number of clusters, *k*, was chosen using the ConsensusClusterPlus method (Wilkerson and Hayes, 2010)

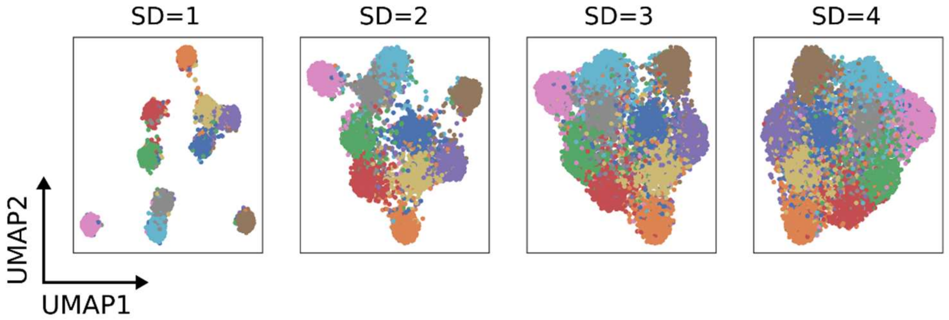

Supplementary Figure S10: UMAP embeddings showing the distribution of ten Gaussian 'clouds' of synthetically generated data points (see Supplementary Methods) with an increasing standard deviation (SD) causing increasing overlap.
